# Agomelatine, venlafaxine, and running exercise effectively prevent anxiety- and depression-like behaviors and memory impairment in restraint stressed rats

**DOI:** 10.1371/journal.pone.0187671

**Published:** 2017-11-03

**Authors:** Sarawut Lapmanee, Jantarima Charoenphandhu, Jarinthorn Teerapornpuntakit, Nateetip Krishnamra, Narattaphol Charoenphandhu

**Affiliations:** 1 Department of Physiology, Faculty of Science, Mahidol University, Bangkok, Thailand; 2 Center of Calcium and Bone Research (COCAB), Faculty of Science, Mahidol University, Bangkok, Thailand; 3 Physiology Division, Preclinical Sciences, Faculty of Medicine, Thammasat University, Pathumthani, Thailand; 4 Department of Physiology, Faculty of Medical Science, Naresuan University, Phitsanulok, Thailand; 5 Institute of Molecular Biosciences, Mahidol University, Nakhon Pathom, Thailand; Radboud University Medical Centre, NETHERLANDS

## Abstract

Several severe stressful situations, e.g., natural disaster, infectious disease out break, and mass casualty, are known to cause anxiety, depression and cognitive impairment, and preventive intervention for these stress complications is worth exploring. We have previously reported that the serotonin-norepinephrine-dopamine reuptake inhibitor, venlafaxine, as well as voluntary wheel running are effective in the treatment of anxiety- and depression-like behaviors in stressed rats. But whether they are able to prevent deleterious consequences of restraint stress in rats, such as anxiety/depression-like behaviors and memory impairment that occur afterward, was not known. Herein, male Wistar rats were pre-treated for 4 weeks with anti-anxiety/anti-depressive drugs, agomelatine and venlafaxine, or voluntary wheel running, followed by 4 weeks of restraint-induced stress. During the stress period, rats received neither drug nor exercise intervention. Our results showed that restraint stress induced mixed anxiety- and depression-like behaviors, and memory impairment as determined by elevated plus-maze, elevated T-maze, open field test (OFT), forced swimming test (FST), and Morris water maze (MWM). Both pharmacological pre-treatments and running successfully prevented the anxiety-like behavior, especially learned fear, in stressed rats. MWM test suggested that agomelatine, venlafaxine, and running could prevent stress-induced memory impairment, but only pharmacological treatments led to better novel object recognition behavior and positive outcome in FST. Moreover, western blot analysis demonstrated that venlafaxine and running exercise upregulated brain-derived neurotrophic factor (BDNF) expression in the hippocampus. In conclusion, agomelatine, venlafaxine as well as voluntary wheel running had beneficial effects, i.e., preventing the restraint stress-induced anxiety/depression-like behaviors and memory impairment.

## Introduction

Stress can cause psychiatric disorders, disability and mortality. Since stress hormone glucocorticoids can induce the release of several neurotransmitters, e.g., serotonin, norepinephrine, and dopamine [1], mood disorders are often intractable to conventional pharmacological treatments that target single neurotransmitter, such as benzodiazepines and fluoxetine [2]. How stress affects brain function is not completely understood. Stimulation of the hypothalamic-pituitary-adrenal (HPA) axis and high levels of glucocorticoids decreased brain size, brain-derived neurotrophic factor (BDNF) production, and neurogenesis in the hippocampus of rodents [3, 4]. Our investigation showed that stress induced depletion of monoamine precursors, malfunction of 5-hydroxytryptamine (HT)/adrenergic receptors, inappropriate increases in activities of serotonin and norepinephrine reuptake transporters, and increased monoamine degradation [5].

The serotonin-norepinephrine-dopamine reuptake inhibitor (SNDRI) venlafaxine has been reported to increase monoamine levels in several brain regions related to anxiety, depression, and memory [6, 7]. Our recent investigation demonstrated that the serotonin-norepinephrine-dopamine reuptake inhibitor (SNDRI), venlafaxine, and voluntary wheel running effectively alleviated anxiety- and depression-like behaviors in stressed male rats [8, 9]. However, besides venlafaxine, agomelatine, the melatonin MT1 and MT2 receptor agonist and 5-HT_2C_ receptor antagonist with robust anxiolytic and antidepressant effects in humans, rats, and mice [10–12], could also be a candidate drug for the preventing stress-induced behavioral change.

Nevertheless, there may be some individuals who do not respond to pharmacological agents; therefore, additional interventions, e.g., exercise, should be beneficial to this group of individuals. Mild-to-moderate intensity exercise has been known to have anxiolytic and antidepressant effects in both humans and rodents [13, 14], but its preventive effect on stress-induced mood disorders is unclear. In stress-free rats, wheel running increased 5-HT_1A_ receptor mRNA level, while decreasing serotonin transporter (SERT) mRNA level in the dorsal raphé, an area responsible for anxiety-like behavior [15, 16]. Voluntary wheel running also activated the dentate gyrus granule neurons and increased hippocampal neurogenesis through upregulation of BDNF mRNA expression [17].

Therefore, the present study aimed *(i*) to determine whether 1-, 4- and 8-week restraint stress could induce anxiety- and depression-like behaviors, and impairments of spatial learning, spatial memory, and novel object recognition in male rats; and (*ii*) to evaluate the effectiveness of venlafaxine, agomelatine, and voluntary wheel running exercise in the reversal of stress-induced sequelae as well as induction of BDNF protein expression in the hippocampus.

## Materials and methods

### Animals

Eight-week-old male Wistar rats (weighing 180–220 g) were obtained from the National Laboratory Animal Center, Mahidol University, Thailand. They were housed in stainless-steel shoebox cages (2 rats/cage) with wire covers (cage dimension 24 × 48 × 18 cm) at 25 ± 2°C and 55 ± 5% humidity under 12:12-h light-dark cycle (light on between 06:00 h and 18:00 h with average illuminance of 200 lux). All rats were fed standard chow (CP Co., Ltd., Thailand) and water ad libitum. After arrival at the vivarium, they were acclimatized for 1 week before the start of the experiments. Regarding alleviation of suffering and euthanasia, all animals were housed in a quiet husbandry unit. They were handled by the same researcher with well-trained gavage skill to minimize handling stress throughout the 1-week acclimatizing and experimental periods. Gavage procedure was gently performed with a gavage tube no. 18, and neither injury nor aspiration was observed. Euthanasia was conducted by applying overdose isoflurane inhalation. Animal care was in accordance with the Guide for the Care and Use of Laboratory Animals, National Research Council (eighth edition). This study has been approved by the Animal Care and Use Committee of the Faculty of Medicine, Thammasat University, Thailand.

In the present study, we used only male rats to avoid female physiological factors (e.g., estrous cycle) that could interfere with data interpretation. Since a decrease in estrogen level can be anxiogenic, female rats are sensitive to anxiety and emotional variability [18, 19]. In addition, some previous studies have reported that male rats exhibited memory loss more than female rats after exposure to chronic stress [20, 21].

### Experimental design

**Fig 1:**
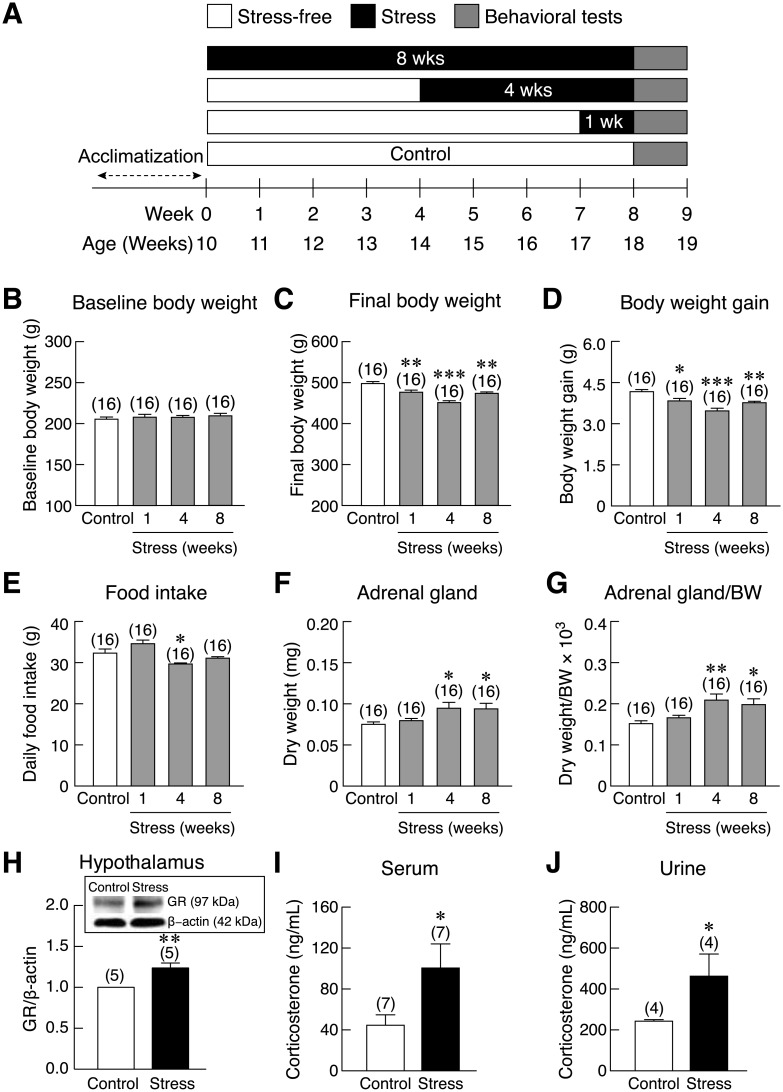
Experimental design and time-dependent changes in the physical and biochemical parameters in restraint stressed rats. (A) Timeline diagram shows restraint stress induction protocol and behavioral tests. All rats were acclimatized for 1 week prior to 1-, 4-, or 8-week stress induction (black). One- and 4-week groups, were subjected to preceding stress-free period of 7 and 4 weeks (gray), respectively. Behavioral tests, i.e., elevated plus-maze (EPM), elevated T-maze (ETM), novel object recognition (NOR), Morris water maze (MWM), and forced swimming test (FST), were performed at the end of stress session. (B) Baseline body, (C) final body weight, (D) weight gain, (E) daily food intake, (F) dry weight of adrenal gland, and (G) dry adrenal gland weight normalized by body weight (BW) in 1-, 4-, and 8-week restraint stressed rats. (H) Glucocorticoid receptor (GR) protein expression normalized by β-actin in control and 1-week stressed rats. *Inset*: representative electrophoresis bands of GR and β-actin. (I–J) Serum and urinary corticosterone levels in 4-week stressed rats. Numbers of animals are noted in parentheses. *p < 0.05, **p < 0.01, ***p < 0.001 stress vs. control.

In the first series of experiments (Fig 1A), rats were divided into 4 groups, i.e., stress-free control group and 3 age-matched stressed groups (n = 16 animals/group), which comprised of stressed rats subjected to restraint stress for 1, 4, or 8 weeks. Body weights and food intake were recorded daily. In 1- and 4-week stressed groups, there was a preceding stress-free period of 7 and 4 weeks, respectively, in order to commence the behavioral tests at the same age. At the end of stress induction, all rats were evaluated for anxiety- and depression-like behaviors, spatial learning, spatial memory, and memory impairment by using elevated plus-maze (EPM), elevated T-maze (ETM), open field test (OFT), forced swimming test (FST), Morris water maze test (MWM), and novel object recognition (NOR). Urine samples were collected from metabolic cage on the last day of stress session using metabolic cage (11:00 am–17:00 pm) to determine corticosterone levels. Then, rats were euthanized 24 h after behavioral tests, and their blood and adrenal glands were collected for measurement of serum corticosterone levels and adrenal weights, the indicators of stress response. However, urinary and serum samples from some rats were inadequate for analyses, and some tissues were not in good condition; therefore, they were excluded from the data sets (numbers of samples are shown in figures). To eliminate diurnal variations of corticosterone levels, the blood samples were always collected in the late morning (8:00 am–11:00 pm).

The second series of experiments was to evaluate the effectiveness of monoaminergic drugs (10 mg/kg body weight agomelatine; Les laboratoires Servier Industrie, Gidy, France), SNDRI (10 mg/kg body weight venlafaxine hydrochloride; Pfizer Ireland pharmaceuticals, Co. Kildare, Ireland), and voluntary wheel running in the prevention of stress-induced anxiety/depression-like behaviors and memory impairment. Rats were divided into 4 groups (n = 16 rats/group), i.e., vehicle (normal saline 5 mL/kg body weight)-, agomelatine-, venlafaxine-, and exercise treated groups. Drugs were administered orally (once-daily for 4 weeks, 7 days/week) via a gavage tube no. 18 to mimic the oral route of drug administration in psychiatric patients. Both drugs and vehicle were freshly prepared daily from commercial tablets, and were administrated at 16:00 h [8]. To determine the beneficial effects of 4-week voluntary exercise under non-stress condition or after 4-week exposure to restraint stress, male rats were divided into non-stressed sedentary, non-stressed voluntary wheel running, stressed sedentary, and stressed and voluntary wheel running (n = 10 rats/group). Heart and adrenal glands were collected and weighed to confirm the effectiveness of exercise protocol and the presence of stress response, respectively. After 4 weeks of pharmacological treatment or exercise intervention, rats were subjected to 4-week restraint stress induction, followed by behavioral tests. Since the results from the first series showed that 8-week stress exposure led to habituation, i.e., less stress response, 4-week stress induction was used in this second part of the experiments.

In all experiments, rats underwent behavioral tests on the day after the end of stress induction (between 8:00–12:00 h). Each rat was subjected to all behavioral tests sequentially, i.e., EPM, ETM, OFT, NOR, MWM, and FST, respectively. EPM, ETM, and OFT were performed on the first day with 5-min intervals between tests. NOR, MWM, and FST were carried out for 2, 3 and 2 days, respectively. As for EPM, ETM, and OFT tests, each rat was brought to the test room (dim light with average illuminance of 20 lux) and left undisturbed in a quiet environment for at least 5 min before EPM was begun, followed by ETM and OFT. Behavioral responses were continuously recorded in dim light by an infrared video camera (model HDR-XR200E; Sony, Tokyo, Japan) installed above the test area. Behavioral apparatus and surrounding area were cleaned after each test with a wet towel and 70% ethanol to eliminate odorants, feces and urine. Each rat was subjected to one test at a time without the presence of other rats in the room. The NOR, MWM, and FST were later conducted as described in the following section.

### Measurement of body, food intake, heart and adrenal weights

Rats were gently removed from their home cages and weighed in grams. Pre-weighed food was provided and 24 h later amount of remaining food was recorded. At the end of the experiment, heart and adrenal glands were removed, washed with ice-cold normal saline solution and blotted dry with a filter paper. Thereafter, the tissues were dried in an incubator at 80°C for 3 days to obtain constant dry weights. The heart weight was used to indicate the effectiveness of exercise protocol.

### Serum samples collection and preparation

Blood samples were collected under isoflurane anesthesia by cardiac puncture. Whole blood was allowed to clot at room temperature for 15 min. Then, the clot was removed by centrifugation (1400 ×g, 15 min, 4°C). Serum was transferred to a new tube and stored at –80°C until measurement of corticosterone levels.

### Measurement of corticosterone levels

The serum and urinary corticosterone levels, indicators of stress response, in control and 4-week stressed rats were determined by commercial enzyme immunoassay kit (catalog no. AC-14F1; Immunodiagnostic Systems Ltd, Tyne and Wear, UK), according to the manufacturer’s instruction, with a detection limit of 0.23 ng/mL with inter- and intra-assay coefficient of variation of <8 and <4%, respectively.

### Protein preparation and western blot analysis

After behavioral test, rats were sacrificed and brain was rapidly removed, frozen in liquid nitrogen, and stored at –80°C. Hypothalamus and hippocampus were isolated according to modified method of Heffner et al. (1980) [22]. In brief, brain tissues were lysed in RIPA buffer supplemented with protease and phosphatase inhibitor cocktails (Sigma, St. Louis, MO, USA), homogenized with a handheld pestle and mortar, sonicated and then centrifuged to collect supernatant protein. Total protein concentration was quantified using BCA Protein Assay kit (Thermo Scientific Inc. Waltham, MA, USA).

Protein samples (50 μg/well) were loaded on 10% sodium dodecyl sulphate polyacrylamide gel electrophoresis and transferred onto nitrocellulose membranes. Thereafter, membranes were incubated with 1:1000 rabbit polyclonal anti-BDNF antibody (catalog no. sc-546, Santa Cruz Biotechnology, CA, USA), 1:1000 mouse monoclonal anti-glucocorticoid receptor (GR) antibody (catalog no. AB2768, Abcam, Cambridge, UK), or 1:2000 mouse monoclonal anti-β-actin antibody (catalog no. sc-47778, Santa Cruz) at 4°C overnight. After washing, membranes were incubated with 1:2000 goat anti-rabbit or goat anti-mouse secondary antibody (catalog no. sc-2004 or sc-2005, Santa Cruz, respectively) at 25°C for 2 h. Protein bands were detected by using enhanced chemiluminescence (ECL Plus; Amersham Biosciences) and visualized under FluorChem SP 4.1 system (Alpha Innotech, San Leandro, CA, USA). Densitometric analysis was performed using ImageJ software.

### Restraint stress induction

The stress protocol conduced in a quiet room between 8:00 h and 10:00 h was modified from the methods of Lapmanee et al. (2013) [8]. Rats in the stressed group were restrained for 2 hours/day, 5 days/week for 1, 4, or 8 weeks. The restraint procedure included immobilizing the rat in a 24 × 6 cm transparent polyethylene terephthalate cylinder fixed with transparent plastic tape. The cylinder had a hole of 1 cm diameter at one end for breathing.

### Voluntary wheel running exercise

Voluntary exercise protocol was modified from the methods of Lapmanee et al. (2013) and Droste et al. (2007) [8, 23]. The running distance was calculated from the number of turns multiplied by the circumference (144 cm) of the running wheel, which was placed in a polycarbonate cage (model 80859; Lafayette Instrument Company, Lafayette, IN, USA). The number of turns was recorded by an electronic counter. During exercise session, each rat was housed in an unlocked running wheel (7 days/week for 4 weeks), whereas non-running (sedentary) rats were placed in cages with locked running wheel. During the stress induction period, rats no longer received pharmacological treatment or exercise intervention.

### Elevated plus-maze test (EPM)

The maze was made of wood painted black and elevated 0.5 m above the floor. The apparatus was composed of two open arms (50 × 10 cm) aligned perpendicularly to two closed arms (50 × 10 × 40 cm). The open arms had 1-cm high Plexiglas rim to prevent fall. A rat was individually placed onto the central square of the maze, facing between open arm and closed arm. It was then allowed to explore the maze for 5 min, which behavioral responses were continuously monitored by an infrared camera. Behavioral parameters determined in the present study were time spent and number of entries into the open and closed arms, and total number of arm entries. Arm entry was recorded only when all four paws entered that arm. An increase in time spent in the open arm, increased number of open arm entries, and/or decreased number of closed arm entries or time indicated anxiolysis [24].

### Elevated T-maze test (ETM)

In this study, ETM test was used to evaluate anxiety based on the study of Graeff et al. (1993) [25]. An apparatus was made from wood painted black, elevated 0.5 m above the floor and composed of three arms with equal dimension of 50 × 10 cm. The closed arm with 40-cm wall was placed perpendicularly to two opposed open arms. These three arms were connected by the 10 × 10 cm central square. The open arms were also guarded by 1-cm high Plexiglas rims to prevent fall. The ETM test consisted of three inhibitory avoidance trials (i.e., baseline, avoidance 1 and avoidance 2) and one-way escape trial (30-s interval). Regarding the inhibitory avoidance trial, a rat was individually placed at the terminal end of the closed arm, facing the central square. Baseline time was determined as the time taken to withdraw from the closed arm with all four paws. The same procedure was repeated for two additional avoidance trials (avoidance 1 and 2 latencies). After the avoidance trials, one escape trial was performed by placing a rat on the terminal end of the right open arm, facing the central square. The escape latency was the time taken to exit this arm and enter the closed arm with four paws. The inhibitory avoidance (both increased avoidance 1 and 2) represented learned or conditioned fear—one of the anxiety-like behaviors—whereas one-way escape represented innate or unconditioned fear [26].

### Open field test (OFT)

The apparatus was made of wood painted black (76 cm long × 57 cm wide × 35 cm high) with a 48-square grid floor (6 × 8 squares, 9.5 cm per side). The arena is divided into two zones, namely inner and outer zones (24 peripheral squares). The animal was gently placed in one of the four corner squares and given 5 min to explore the apparatus. Behavioral responses were recorded by infrared video camera. An increase in time spent in the inner zone or a decrease in time spent in the outer zone (i.e., a reduction in thigmotaxis) indicated anxiolysis or increased exploration. Change in the number of lines crossed—number of lines crossed in the first 30 s and total lines crossed—represented change in locomotor activity [26].

### Novel object recognition (NOR)

The procedure was modified from the methods of Redrobe et al. (2010) [27]. NOR was performed in a black rectangular plastic box (63 cm long × 63 cm wide × 45 cm high) in 360 lux room light. A video camera was installed on a movable trolley above the box to record behavior. The objects to be discriminated were made of glass or ceramic. On the day before the NOR test, each rat was allowed to habituate the empty box (2 sessions, 10 min/session). During the test, each rat was gently placed into the box and exposed for 3 min to an acquisition session with identical objects (two pepper ceramic bottles; 3 cm long × 3 cm wide × 7 cm high), ~10 cm apart at the center of the box. The rat was then transferred to its cage for 60 min. Meanwhile, the box and objects were cleaned. One object in the box was replaced with a novel object (glass paperweight; 5 cm long × 5 cm wide × 12 cm high). The same rat was then returned to the box and allowed to explore the new object for 3 min. Object-exploring behaviors included sniffing, licking, or touching each object. The discrimination ratio was calculated from the following equation.

Discriminationratio=(timeexploringnovelobject−timeexploringfamiliarobject)/totalexplorationtime

A decrease in discrimination ratio indicated cognitive and memory impairment in stressed rats [27, 28].

### Morris water maze test (MWM)

Learning and memory were evaluated by using MWM, which was modified from the methods of Morris et al. (1981) [29] and Carman & Mactutus (2002) [30]. The maze was a circular pool made of stainless steel (150 cm diameter and 60 cm height), filled with water (31 cm depth) maintained at 25°C. Water was made opaque with 200 mL milk. Each rat was gently placed in one of the four quadrants (North, South, East, and West) at the start of each trial. A stainless steel platform (diameter of 10 cm, 30 cm in height) was placed 1 cm beneath the surface of the water. Lighting in the room was arranged to provide even illumination in all quadrants. The spatial visual cues consisting of different shapes and colors (i.e., black/white circle and cross and black grid) were visible on each wall of the room to provide orientation during the navigational learning trials and memory probe test.

Prior to learning trials, a rat was individually placed on the platform for 10 s to familiarize it to the task. When the rat moved away from the platform before the end of 10 s, it was placed back onto the platform. This orientation procedure was repeated 3 times, and the learning trials were performed immediately thereafter. Each rat was placed in different location (North, South, East, or West) near the edge of the pool with its front paws first touching the wall. Then, it was allowed to find the hidden platform, and time spent for locating the platform (escape latency) was noted. The hidden platform was placed at a fixed location, i.e., in the Southeast quadrant. When the rat was unable to locate the platform within 60 s, it was placed on the platform and an escape latency of 60 s was recorded. Finally, the rat was given 10 s to remain on the platform to familiarize itself with the surrounding visual cues. Rats performed 8 trials on day 1 and 2, and 4 trials on day 3 (total 20 trials), according to the method of Morris (1981) [29]. There was a 5-min inter-trial interval [30], during which each rat was kept warm in a cage under a heating lamp.

Spatial memory was evaluated by a probe test 1 h after the last learning trial on day 3. Rat was individually placed in the North quadrant in the absence of a platform. Each rat was allowed to swim for 60 s, and time spent in each quadrant was recorded by a video camera. An increase in escape latency indicated poor spatial learning, whereas an increase in correct quadrant time (i.e., time to reach the correct quadrant) indicated spatial memory impairment.

### Forced swimming test (FST)

Each rat was individually forced to swim in a cylinder (45 cm high, 25 cm diameter) filled with 25°C tap water up to the depth of 35 cm. FST consisted of two tests in a 24-h period. In the first swimming session, rat was placed in the water for 15-min assessment. Thereafter, the animal was removed from water, dried, and cleaned with a towel before returned to the cage. In the next swimming session, each rat was placed back in the swimming cylinder for 5 min. Duration of immobility behavior (floating in water with only movement necessary to keep the head above water), swimming behavior (active movement of the forepaws with goal-directed horizontal actions, such as crossing between quadrants of the cylinder and turning), and climbing behavior (upward goal-directed movements of the forepaws along the wall of the cylinder) were recorded. An increase in immobility duration or decreases in swimming duration or climbing duration indicated depression-like behaviors [31].

### Statistical analyses

The results were expressed as means ± SE. Comparisons between the two data sets were performed by unpaired Student’s *t*-test. Multiple comparisons were analyzed by one-way analysis of variance (ANOVA) with Dunnett’s multiple comparison test. The *t*-values, *F*-values, degree of freedom (df), and *p*-values were also presented. The level of significance was *p* < 0.05. All tests were analyzed by GraphPad Prism 6.0 (GraphPad Software Inc., San Diego, CA, USA).

## Results

### Restraint stress induced anxiety- and depression-like behaviors and memory impairment

Baseline body weight was similar among four groups of experiment (Fig 1B). After 1-, 4-, and 8-week restraint stress induction, the final body weights of stressed rats were significantly lower (Fig 1C), leading to less body weight gain as compared to control rats [*F*(3,60) = 13.487, *p* < 0.001] (Fig 1D). Daily food intake was reduced only in the 4-week stress group compared with control group [*F*(3,60) = 9.337, *p* < 0.001] (Fig 1E). Restraint stress (4 and 8 week) led to increased dry adrenal gland weight [*F*(3,60) = 3.684, *p* = 0.017] and relative adrenal gland weight [*F*(3,60) = 5.637, *p* = 0.002] (Fig 1F and 1G), an indicator of successful stress induction. Hypothalamic glucocorticoid receptor (GR) protein expression was significantly higher in 1-week stressed rats than that of the control rats [*t*(8) = 3.990, *p* = 0.002] (Fig 1H), indicating hyperactivity of HPA axis. In addition, serum and urinary corticosterone levels in 4-week stressed rats were also increased [*t*(12) = 2.142, *p* = 0.027; *t*(6) = 2.162, *p* = 0.034] (Fig 1I and 1J), consistent with the adrenal weight.

**Fig 2:**
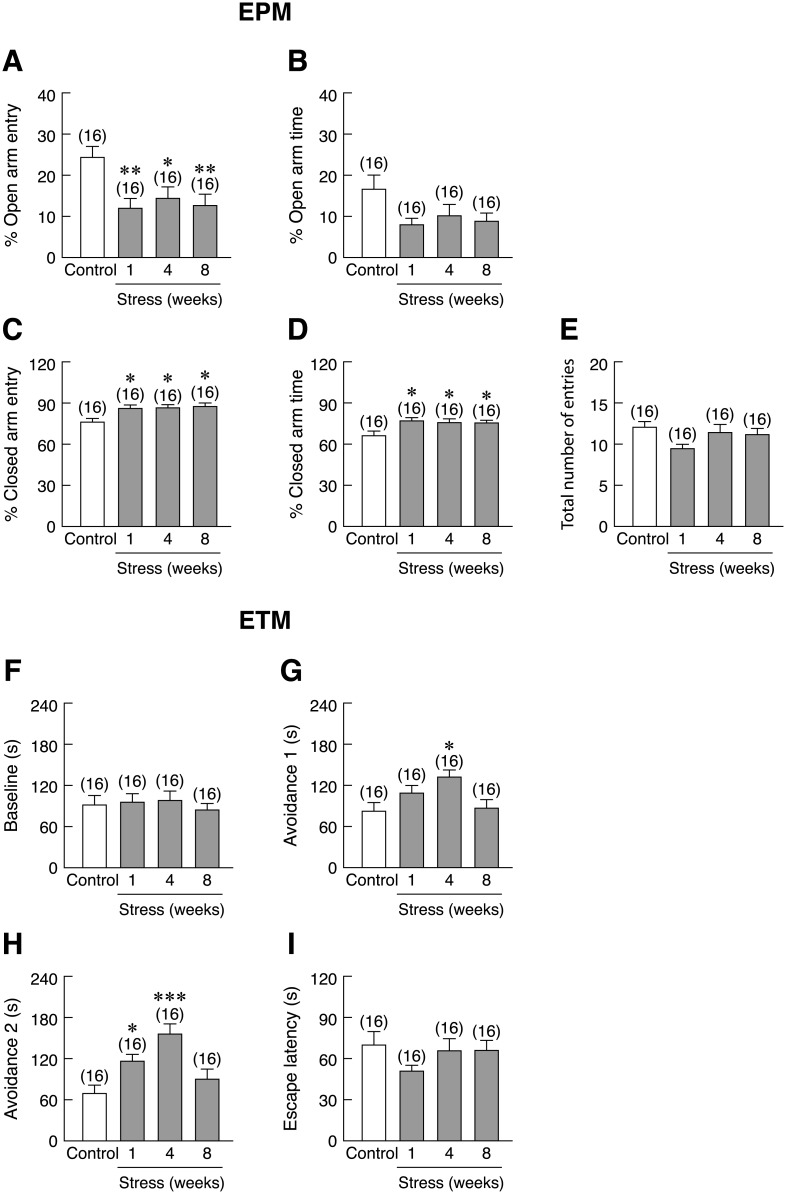
Time-dependent changes in the stress-induced anxiety-like behaviors in rats as determined by EPM and ETM. (A) Percent open arm entry, (B) percent open arm time, (C) percent closed arm entry, (D) percent closed arm time, and (E) total number of entries in 1-, 4-, and 8-week stressed male rats, as determined by elevated plus-maze (EPM). (F) One-way escape latency, (G) baseline time, (H) avoidance 1, and (I) avoidance 2 in stressed rats, as determined by elevated T-maze (ETM). Numbers of animals are noted in parentheses. *p < 0.05, **p < 0.01, ***p < 0.001 stress vs. control.

**Fig 3:**
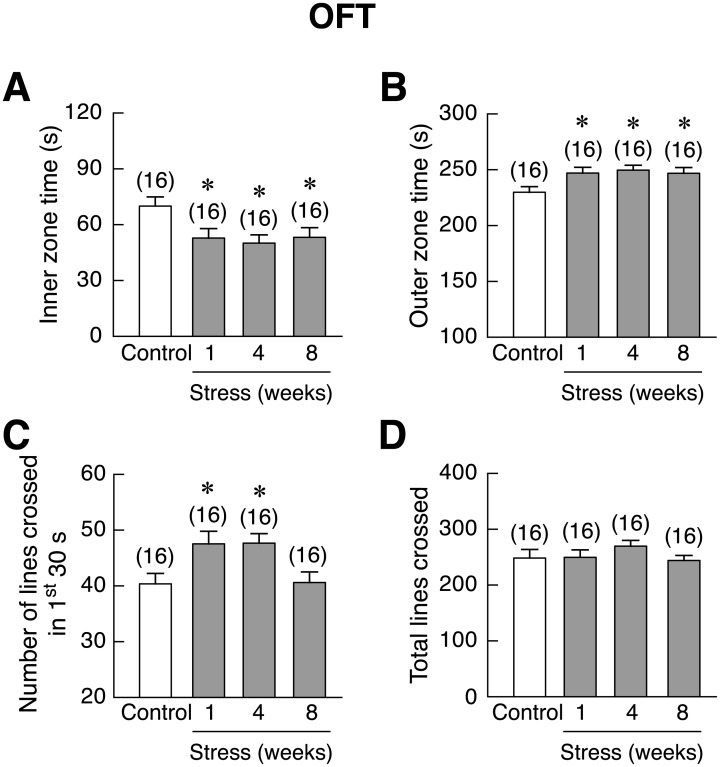
Time-dependent changes in the stress-induced anxiety-like behaviors in rats as determined by OFT. (A) Inner zone time, (B) outer zone time, (C) number of lines crossed in the first 30 seconds, and (D) total lines crossed in 1-, 4-, and 8-week stressed male rats, as determined by open field test (OFT). Numbers of animals are noted in parentheses. *p < 0.05 stress vs. control.

Restraint stressed rats manifested anxiety-like behavior, as demonstrated by EPM and ETM tests (Fig 2). In EPM study, 1-, 4-, and 8-week restraint stress resulted in decreased open arm entry [*F*(3,60) = 4.537, *p* = 0.006] (Fig 2A), increased closed arm entry [*F*(3,60) = 3.860, *p* = 0.014] and closed arm time [*F*(3,60) = 3.504, *p* = 0.021] without change in the total number of entries (Fig 2C–2E). Restraint stress also had tendency to decrease open arm time [*F*(3,60) = 2.406, *p* = 0.076] (Fig 2B). In the ETM test, restraint stress did not significantly alter the one-way escape latency or the baseline time of avoidance test (Fig 2F and 2G). Interestingly, 4-week stress led to greater avoidance 1 [*F*(3,60) = 3.747, *p* = 0.016], while 1- and 4-week, but not 8-week stress, showed greater avoidance 2 [*F*(3,60) = 7.977, *p* < 0.001] (Fig 2H and 2I). In the OFT test, stressed rats spent less time in the inner zone or more time in the outer zone [*F*(3,60) = 3.423, *p* = 0.023] (Fig 3A and 3B). The 1- and 4-week stressed rats were hyperarousal to the sudden change from a familiar environment to an open area as shown by an increase in the number of lines crossed in the first 30 seconds [*F*(3,60) = 4.550, *p* = 0.006] (Fig 3C) but no changes in total lines crossed in 5 min [*F*(3,60) = 0.865, *p* = 0.465] (Fig 3D).

**Fig 4:**
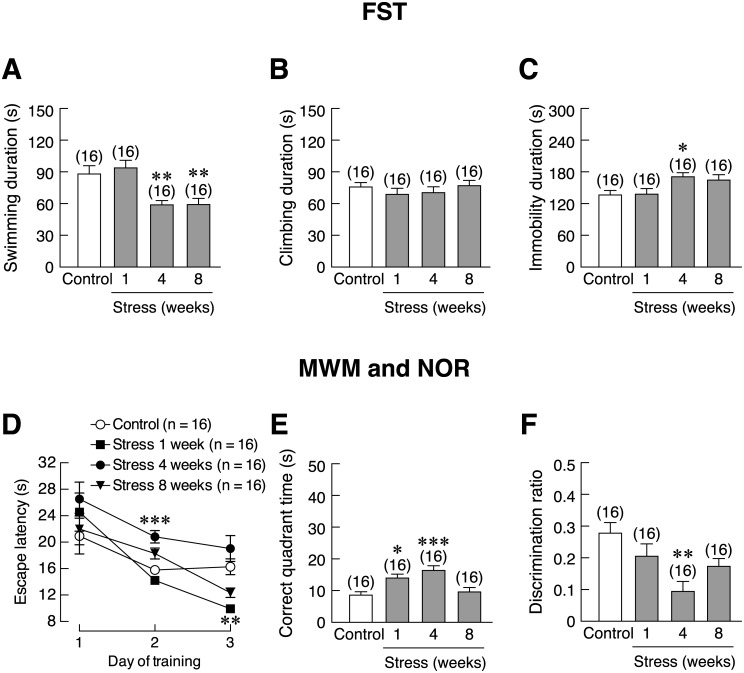
Time-dependent changes in the stress-induced depression-like behaviors and memory impairment in rats. (A) Swimming duration, (B) climbing duration, and (C) immobility duration in 1-, 4-, and 8-week stressed male rats, as determined by forced swimming test (FST). (D) Escape latency, (E) correct quadrant time, and (F) discrimination ratio in stressed rats, as determined by Morris water maze (MWM) and novel object recognition (NOR) tests. Numbers of animals are noted in parentheses. *p < 0.05, **p < 0.01, ***p < 0.001 stress vs. control.

Besides inducing anxiety-like behavior, restraint stress reduced swimming duration (4- and 8-week) [*F*(3,60) = 7.759, *p* < 0.001] and increased immobility duration [*F*(3,60) = 3.493, *p* = 0.021] without changes in climbing duration in FST, suggesting that stress could induce depression-like behavior (Fig 4A–4C). In the MWM that was used to evaluate learning and memory, 4-week stressed rats showed an increase in escape latency on day 2 of the test [*F*(3,28) = 13.864, *p* < 0.001], whereas on day 3, only the escape latency of the 1-week stressed rats was lower than that of control [*F*(3,12) = 10.621, *p* = 0.001] (Fig 4D). Correct quadrant time (time spent to find the correct quadrant) was greater than in control rats in 1- and 4-week stressed rats [*F*(3,60) = 8.225, *p* < 0.001], indicating spatial memory impairment (Fig 4E). Finally, the NOR test showed that 4-week stressed rats demonstrated reduced discrimination ratio as compared to that of control group [*F*(3,60) = 5.446, *p* < 0.001] (Fig 4F), suggesting impaired cognitive and memory.

### Exercise and pharmacological treatments effectively prevented anxiety-like behaviors

**Fig 5:**
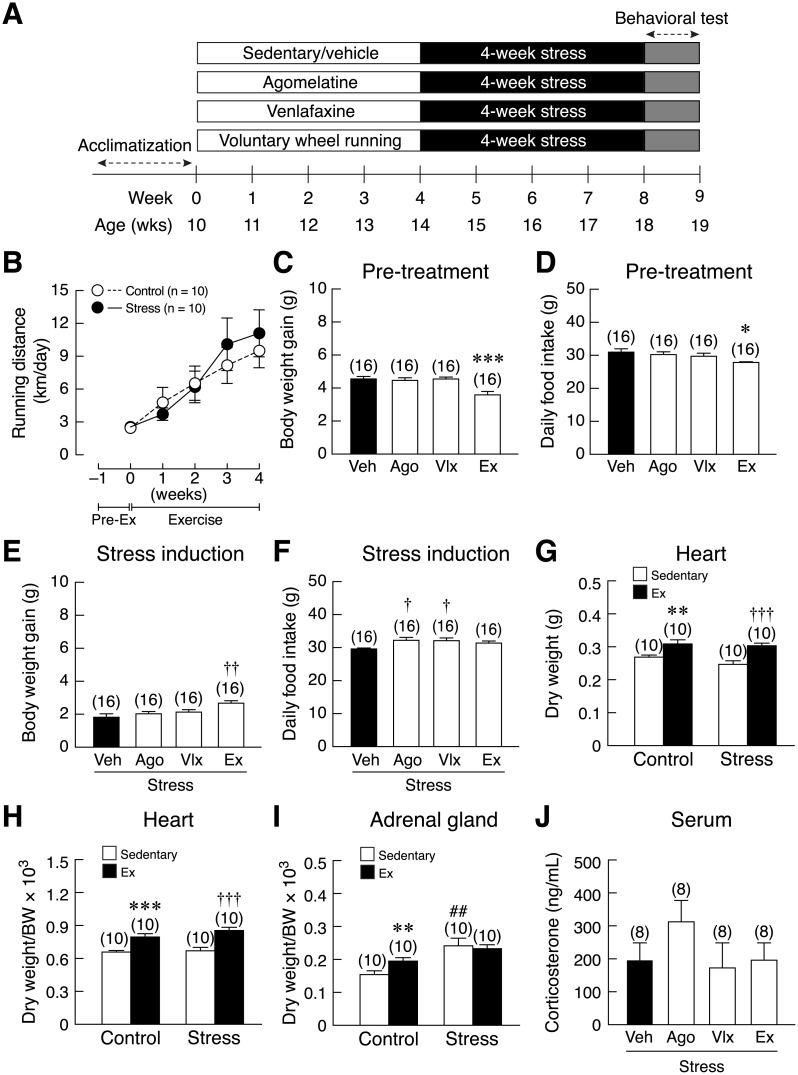
Experimental design and the effects of pharmacological treatments and voluntary wheel running on physical and biochemical parameters in stressed rats. (A) Timeline diagram shows voluntary wheel running and pharmacological treatment protocols. After 1-week acclimatization, rats were divided into one control (vehicle-treated (Veh)/sedentary) and three experimental groups, i.e., agomelatine-treated (Ago), venlafaxine-treated (Vlx), and running (Ex) groups. Drug administration and exercise intervention were given for 4 weeks (pre-treatment), followed by 4-week stress exposure (stress induction), during which rats received neither drug treatment nor exercise intervention. Behavioral tests were performed at the end of stress induction period. (B) Running distance per day in control and stressed groups. Body weight gain and daily food intake, after pre-treatment (C–D, respectively) and after stress induction period (E–F, respectively). (G) Dry adrenal gland weight normalized by BW, (H) dry heart weight, and (I) dry heart weight normalized by body weight (BW) in sedentary and running rats. The control group was stress-free, whereas the stress group was exposed to 4-week restraint stress. (J) Serum corticosterone levels in stressed rats subjected to each treatment. Pre-Ex, pre-exercise period. Numbers of animals are noted in parentheses. *p < 0.05, **p < 0.01, ***p < 0.001 exercise group vs. control (Veh/sedentary) group. ^†^*p* < 0.05, ^††^*p* < 0.01, ^†††^*p* < 0.001 each experimental group vs. stressed (Veh/sedentary) group. ^##^*p* < 0.01 stressed sedentary group vs. sedentary control group.

To determine the protective effects of pharmacological treatments and voluntary wheel running on anxiety- and depression-like behaviors, either running or pharmacological treatment was given for 4-week prior to stress induction (Fig 5A). In the running experiment, running distances were the same in control and stressed rats (Fig 5B). After 4-week pre-treatment, voluntary wheel running rats had lower daily body weight gain [*F*(3,60) = 8.281, *p* < 0.001] and food intake [*F*(3,60) = 2.787, *p* = 0.048] compared with other groups (Fig 5C and 5D). Thereafter, 4-week stress induction was applied in all groups, which resulted in high weight gain in running rats compared with stress sedentary rats [*F*(3,60) = 2.828, *p* = 0.046] (Fig 5E) and increased daily food intake was observed only in agomelatine or venlafaxine groups [*F*(3,60) = 4.967, *p* = 0.004] (Fig 5F). For both control and stressed rats, voluntary exercise significantly increased dry heart weights [*t*(18) = 2.969, *p* = 0.004; *t*(18) = 3.940, *p* < 0.001] (Fig 5H) and dry heart weights normalized by body weight [*t*(18) = 4.255, *p* < 0.001; *t*(18) = 4.452, *p* < 0.001] (Fig 5I), indicating that the present exercise protocol effectively induced cardiac hypertrophy—an adaptive cardiovascular response to exercise. Regarding adrenal gland dry weight, increase in adrenal weight normalized by body weight was observed in stressed rats [*t*(18) = 3.254, *p* = 0.002]. Moreover, running control had greater relative adrenal dry weight than the sedentary control rats [*t*(18) = 2.687, *p* = 0.008] (Fig 5G). Neither voluntary running nor drugs (agomelatine or venlafaxine) altered serum corticosterone levels as compared to vehicle-treated sedentary stressed rats (Fig 5J).

**Fig 6:**
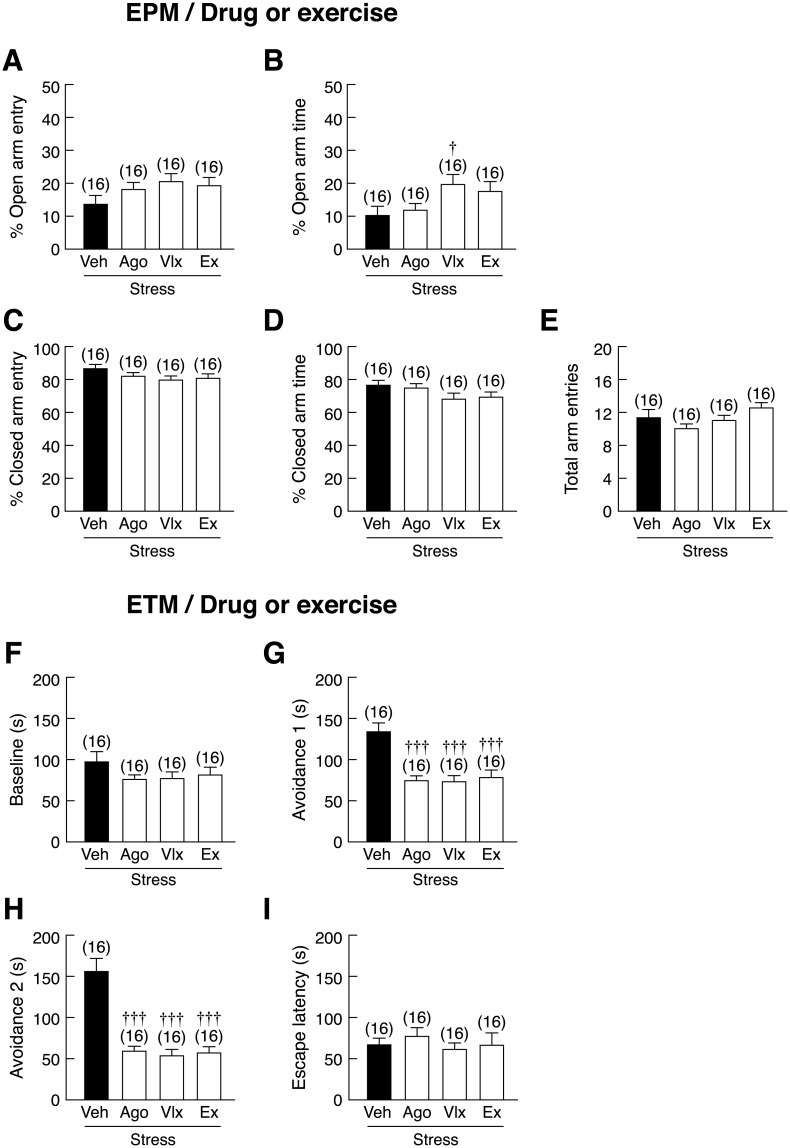
Effects of pharmacological treatments and voluntary wheel running on anxiety-like behaviors in stressed rats. (A) Percent open arm entry, (B) percent open arm time, (C) percent closed arm entry, (D) percent closed arm time, and (E) total arm entries in stressed rats subjected to agomelatine (Ago) or venlafaxine (Vlx) treatment or voluntary wheel running (Ex), as determined by elevated plus-maze (EPM). (F) One-way escape latency, (G) baseline, (H) avoidance 1, and (I) avoidance 2 in stressed rats as determined by elevated T-maze (ETM). Numbers of animals are noted in parentheses. ^†^*p* < 0.05, ^†††^*p* < 0.001 compared with vehicle (Veh)-treated group.

**Fig 7:**
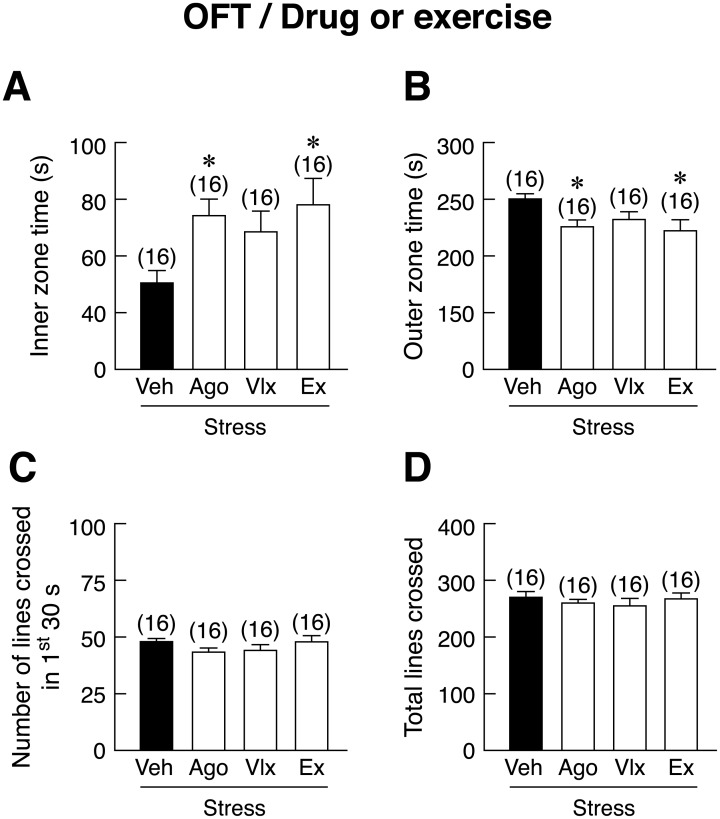
Effects of pharmacological treatments and voluntary wheel running on anxiety-like behaviors in stressed rats. (A) Inner zone time, (B) outer zone time, (C) number of lines crossed in the first 30 seconds, and (D) total lines crossed in stressed rats subjected to agomelatine (Ago) or venlafaxine (Vlx) treatment or voluntary wheel running (Ex), as determined by open field test (OFT). Numbers of animals are noted in parentheses. *p < 0.05 compared to vehicle (Veh)-treated group.

In EPM test, pre-treatment with venlafaxine, but not agomelatine, or voluntary wheel running significantly increased percent open arm time in stressed rats as compared to vehicle-treated stressed rats [*F*(3,60) = 2.767, *p* = 0.050] (Fig 6B). However, neither pharmacological treatments nor running altered percent open arm entry, percent closed arm entry, percent closed arm time, or total arm entries (Fig 6). In the ETM test, no effect on the one-way escape latency or baseline time was observed (Fig 6F and 6G). Agomelatine, venlafaxine, and voluntary wheel running significantly reduced avoidance 1 [*F*(3,60) = 11.820, *p* < 0.001] and avoidance 2 [*F*(3,60) = 26.609, *p* < 0.001] (inhibitory avoidance or learned fear, anxiety-like behavior) in stressed rats compared with vehicle-treated stressed rats (Fig 6H and 6I). To confirm that voluntary wheel running and pharmacological treatments did have a preventive effect on stress-related behaviors, the OFT was also performed. The results showed that exercising rats and agomelatine-, but not venlafaxine-treated rats spent more time in the inner zone of open arena and less time in the outer zone [*F*(3,60) = 3.107, *P* = 0.033] (Fig 7A and 7B), suggesting that running effectively prevented stress-induced anxiety in male rats. No change in number of lines crossed in 1^st^ 30s and total lines crossed was observed among these groups (Fig 7C and 7D).

### Pharmacological treatments, but not voluntary wheel running, successfully prevented depression-like behavior in stressed rats

**Fig 8:**
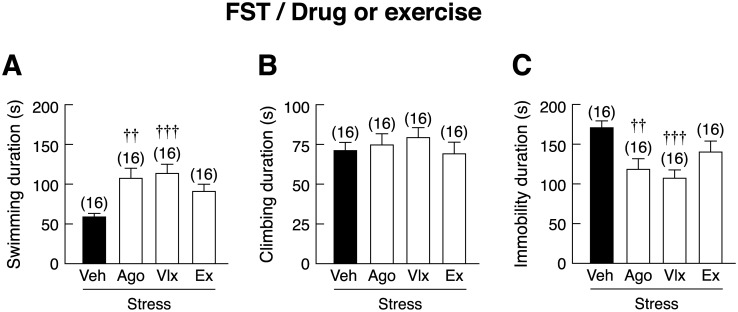
Effects of pharmacological treatments and voluntary wheel running on the prevention of depression-like behavior in stressed rats. (A) Swimming duration, (B) climbing duration, and (C) immobility duration in stressed rats subjected to agomelatine (Ago) or venlafaxine (Vlx) treatment or voluntary wheel running (Ex), as determined by forced swimming test (FST). Numbers of animals are noted in parentheses. ^††^*p* < 0.01, ^†††^*p* < 0.001 compared with vehicle (Veh)-treated group.

Although voluntary wheel running was found to effectively prevent anxiety-like behavior, it was apparently not effective for reducing stress-induced depression-like behavior, as indicated by no changes in swimming duration, climbing duration, or immobility duration in FST as compared to vehicle-treated stressed rats (Fig 8A–8C). On the other hand, both agomelatine- and venlafaxine-treated stressed rats showed longer swimming duration [*F*(3,60) = 6.201, *p* = 0.001] and less immobility duration than vehicle-treated stressed rats [*F*(3,60) = 5.633, *p* = 0.002] (Fig 8A and 8C).

### Both pharmacological treatments and voluntary wheel running improved memory in stressed rats

**Fig 9:**
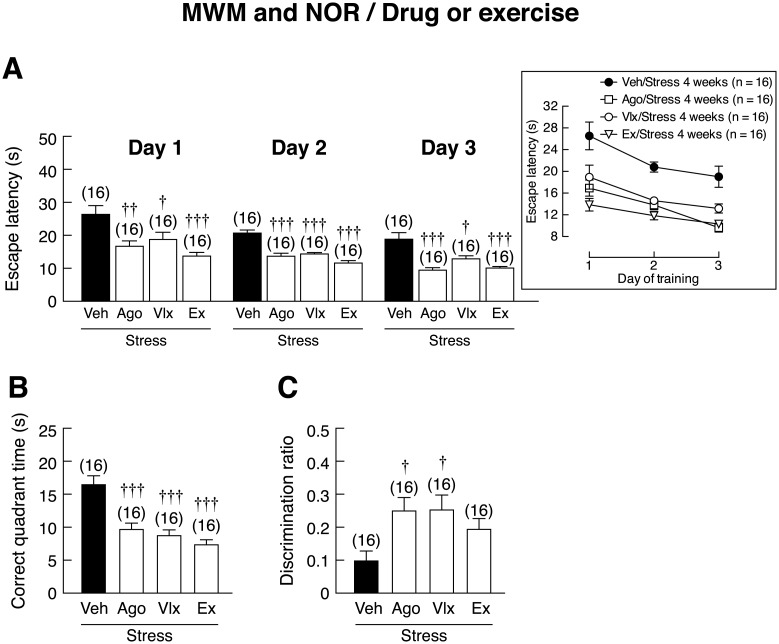
Effects of pharmacological treatments and voluntary wheel running on memory impairment in stressed rats. (A) Escape latency and (B) correct quadrant time in stressed rats subjected to agomelatine (Ago) or venlafaxine (Vlx) treatment or voluntary wheel running (Ex), as determined by Morris water maze (MWM). (C) Discrimination ratio for novel object recognition (NOR) in stressed rats. Numbers of animals are noted in parentheses. ^†^*p* < 0.05, ^††^*p* < 0.01, ^†††^*p* < 0.001 compared with vehicle (Veh)-treated group.

**Fig 10:**
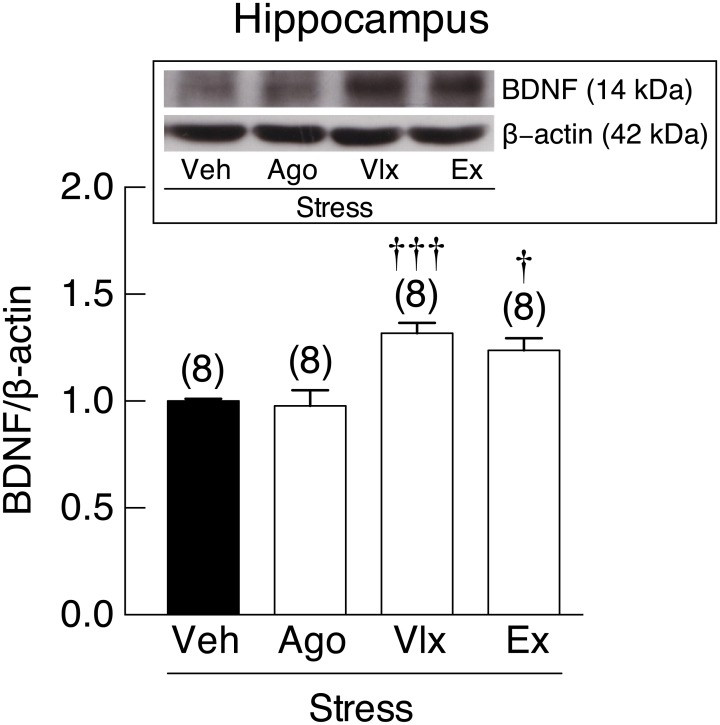
Expression of brain-derived neurotrophic factor (BDNF) in stressed rats pre-treated with drugs and exercise. Hippocampal BDNF protein expression normalized by β-actin in 4-week stressed rats subjected to agomelatine (Ago) or venlafaxine (Vlx) treatment or voluntary wheel running (Ex) as determined by Western blot analysis. *Inset*: representative electrophoresis bands of BDNF and β-actin. Numbers of animals are noted in parentheses. ^†^*p* < 0.05, ^†††^*p* < 0.001 compared with vehicle (Veh)-treated group.

MWM revealed that the agomelatine-, venlafaxine-, and exercise-treated stressed rats all exhibited reduction in escape latency on day 1 [*F*(3,28) = 7.762, *p* < 0.001], day 2 [*F*(3,28) = 24.539, *p* < 0.001] and day 3 [*F*(3,12) = 13.592, *p* < 0.001] and correct quadrant time [*F*(3,60) = 15.790, *p* <0.001] when compared to vehicle-treated stressed rats (Fig 9A and 9B). On each studied day, drug- and exercise-treated groups showed less escape latency than vehicle-treated sedentary group, suggesting that drugs and exercise had protective effects against stress-induced learning and memory impairment. Improvement of novel object recognition was evidenced by higher discrimination ratio in agomelatine- and venlafaxine-treated rats than vehicle-treated stressed rats [*F*(3,60) = 3.647, *p* = 0.018]. However, the exercise- and vehicle-treated rats showed similar discrimination ratio (Fig 9C). Western blot analysis also revealed that pre-treatment with venlafaxine and running exercise were able to upregulate BDNF protein expression by 31.81% and 23.50%, respectively, in the rat hippocampus [*F*(3,28) = 10.520, *p* < 0.001] (Fig 10).

## Discussion

Restraint stress profoundly worsened several aspects of the brain function in male rats, as indicated by the presence of anxiety- and depression-like behavior as well as impairment of spatial learning, spatial memory, and novel object recognition. Adrenal hyperplasia and elevated corticosterone levels confirmed the effectiveness of stress induction [32]. Although reduction in food intake during stress could lead to weight loss, reduced weight seen in all stressed groups was likely to result from corticosterone-induced catabolic state and muscle wasting [33]. In addition, the absence of change in adrenal gland weight of the 1-week stressed rats suggested that 1-week duration was too short to have long-lasting effect.

Based on the EPM and the OFT tests, the restraint stressed rats appeared to develop anxiety-like behavior. The ETM findings of the 1- and 4-week stressed rats further supported the presence of learned (conditioned) fear, but not innate fear, suggesting that the restraint stressed rats had generalized anxiety disorder-like condition without panic [8, 9]. Besides anxiety-like behavior, 4- and 8-week stressed rats demonstrated depression-like behavior as indicated by the decreased swimming duration or increased immobility duration. Since the previous pharmacological study suggested that noradrenergic neurotransmission mediated climbing whereas swimming in FST was associated with serotonergic neurotransmission [16], the restraint stress in the present study predominantly affected serotonin-related mechanism. Interestingly, some behavioral responses observed in 1- or 4-week stressed groups were absent in the 8-week stressed group. The exact explanation for this phenomenon is unclear, but it might be explained in terms of habituation to prolonged restraining procedure.

Behavioral changes, especially impairment of learning and memory, were more complicated. As shown in the MWM test, an increase in escape latency—an indicator of impaired spatial learning—was observed in 4-week stressed rats (day 2), but the spatial learning was apparently improved in 1-week stressed rats (day 3). This was consistent with the previous report that acute exposure to mild-to-moderate stress led to better learning in radial arm maze test [21]. However, the spatial memory or ability to retrieve previous consolidated information, as indicated by an increase in time spent to reach correct quadrant, was found to be impaired in 1- and 4-week stressed rats, whereas the NOR test showed 4-week stressed rats having poor ability to discriminate a previously explored object from a new object, a sign of memory impairment [27, 28].

The underlying mechanisms by which restraint stress led to aberrant behaviors and memory impairment are not completely understood. It is possible that most of the stress-induced sequelae are mediated by elevated levels of corticosterone, which can traverse the blood-brain barrier into the brain [34]. Sairanen et al. (2007) reported that the principal glucocorticoid-responsive brain regions were the prefrontal cortex, hippocampus, and amygdala, all of which are known to modulate anxiety, depression, learning, and memory [35]. Furthermore, stress and glucocorticoid exposure could alter central monoamine levels [1]. For instance, chronic stress reportedly increased norepinephrine metabolite, 3-methoxy-4-hydroxyphenylglycol (MHPG), in the mouse hypothalamus and hippocampus, suggesting an increase in norepinephrine synthesis [36]. Increased levels of dopamine metabolite, 3,4-dihydroxyphenylacetic acid (DOPAC), were reported in the rat frontal cortex, hypothalamus, hippocampus, and amygdala [37]. Similarly, the levels of serotonin and its metabolite, 5-hydroxyindoleacetic acid (5-HIAA), were increased in the rat frontal cortex, nucleus accumbens, hypothalamus, and amygdala [37]. In addition, corticosterone decreased 5-HT_1A_ receptor expression in the dentate gyrus [38] and inhibited serotonin transport via organic cation transporters in the rat hypothalamus [39].

Activation of the HPA axis can also modulate melatonin rhythm, thus melatonin production and secretion pattern. There was a reduction in melatonin secretion in the dark phase after stress exposure, which probably resulted from a reduction in tryptophan precursor for melatonin synthesis. However, nocturnal illumination (2,500 lux, from 20:00 to 06:00 h) did not suppress melatonin production in stressed animals [40]. Corticosterone apparently alters serotonin and melatonin synthesis by modulating the mRNA levels of tryptophan hydroxylase [41]. Although corticosterone reportedly inhibited nuclear factor-κB translocation, thereby enhancing the norepinephrine-induced synthesis of melatonin in the pineal gland [42], chronic stress indirectly impaired sympathetic inputs to the pineal gland, leading to disruption of melatonin rhythm [43]. It was, therefore, possible that derangement of melatonergic system in stressed rats might have stemmed from inappropriate melatonin production and its irregular rhythm.

The aforementioned findings clearly showed the interrelation between chronic stress, sympathetic activity, melatonin system, and several monoaminergic neurotransmissions (norepinephrine, dopamine, and serotonin). This led to our hypothesis that targeting intervention to protect against stress-induced anxiety, depression, and memory impairment should utilize SNDRI, melatonergic modulator, or exercise intervention that are known to modulate these monoaminergic targets [6, 10, 44]. Herein, both pharmacological treatments (agomelatine and venlafaxine) and voluntary wheel running successfully prevented anxiety-like behavior, especially the learned fear (i.e., generalized anxiety disorder-like), as well as impairment of spatial learning and memory in restraint stressed rats. Drugs and exercise had no effect on locomotor activity in the EPM. However, only pharmacological treatments, but not voluntary wheel running, showed a protective action against depression-like behavior and impaired novel object recognition in restraint stressed rats, even though running concurrently with stress induction could reduce depression-like behaviors [8]. The finding of agomelatine-induced reduction in depression-like behavior was consistent with the previous reports using other behavior tests. For example, Païzanis et al. (2010) reported that, after agomelatine treatment, immobility was significantly reduced in mice subjected to the tail suspension test [45].

Since the beneficial actions of studied drugs and exercise occurred without changes in serum corticosterone levels (Fig 5J), it was plausible that agomelatine, venlafaxine, and exercise might have already induced a long-lasting adaptations in monoaminergic neurotransmission or even neurogenesis in the brain, which lasted the entire stress induction periods. For instance, agomelatine has been known to stimulate cell proliferation within the subgranular layer of the dentate gyrus of rodents [46]. Venlafaxine also enduringly modulated the monoaminergic neurotransmission by increasing serotonin, dopamine and norepinephrine levels in the prefrontal cortex and striatum [47], and increasing the expression of BDNF protein in the rat hippocampus [48]. BDNF possibly promoted the function and survival of dopaminergic, GABAergic, noradrenergic, and serotonergic neurons [49]. Improvement of memory by venlafaxine and running exercise could be explained by increased BDNF expression in the hippocampus [50, 51], which is one of the utmost important brain structures for learning and memory. Furthermore, voluntary wheel running exhibited a selective serotonin reuptake inhibitor (SSRI)-like action [8, 52]. Specifically, exercise increased serotonin levels in the synaptic cleft, thereby potentiating serotonergic neurotransmission [53]. During exercise, norepinephrine in the cell body and its metabolites were also increased in the pons, medulla oblongata, and spinal cord of rats [44]. The mRNA expression of 5-HT_1B_ and α1β adrenergic receptors was upregulated in the locus coeruleus and dorsal raphé [15], which could, in turn, alter adrenergic and serotonergic activities, respectively.

In conclusion, although stress causes derangement of brain monoamine metabolism and monoaminergic neurotransmission [1], pharmacological pre-treatments and exercise, which induce long-lasting adaptations in several monoaminergic systems [15, 47, 54], could effectively prevent the stress-induced anxiety- and depression-like behaviors and memory impairment, the latter of which could be explained by hippocampal BDNF protein expression. However, further investigation is required to demonstrate other cellular and molecular mechanisms by which agomelatine, venlafaxine, and voluntary wheel running reduce the stress sequelae. Finally, the present finding have suggested that, in a situation in which future exposure to stress is anticipated, the melatonergic modulator, SNDRI, and voluntary moderate-intensity exercise could be useful in the prevention against development of mood disorders and memory impairment. Exercise appears to be an ideal low-cost intervention for both prevention and treatment of anxiety in stressed individuals.
